# *frpr-18,* a neuropeptide receptor, regulates organismal lifespan and stress tolerance in *C. elegans*

**DOI:** 10.17912/micropub.biology.000840

**Published:** 2023-06-12

**Authors:** Hafsa Ouaakki, Heetanshi Joshi, Laxmi Rathor, Sung Min Han

**Affiliations:** 1 Physiology and Aging, University of Florida, Gainesville, Florida, United States

## Abstract

The mechanisms underlying neuropeptide signaling regulation of lifespan in *Caenorhabditis elegans* ( *C. elegans* ) remain unclear. FRPR-18 is a mammalian orexin/hypocretin-like receptor and modulates *C. elegans* arousal behavior by acting as a receptor for FLP-2 neuropeptide signaling, which is also associated with the systemic activation of the mitochondrial unfolded protein response (mitoUPR). Here we report our preliminary findings on the role of the *frpr-18* gene in regulating lifespan and healthspan parameters, including stress resistance. Our results showed that *frpr-18(ok2698)* null mutants had a shorter lifespan and reduced survivability against thermal stress and paraquat treatment. On the other hand, loss of *flp-2* function did not affect lifespan or paraquat tolerance but was necessary for normal thermal stress tolerance. These findings suggest that *frpr-18* could play a role in regulating lifespan and stress resistance, possibly through *flp-2* independent or parallel neuropeptide signaling pathways.

**Figure 1. Alterations in longevity and stress tolerance in frpr-18(ok2698) null mutants:**
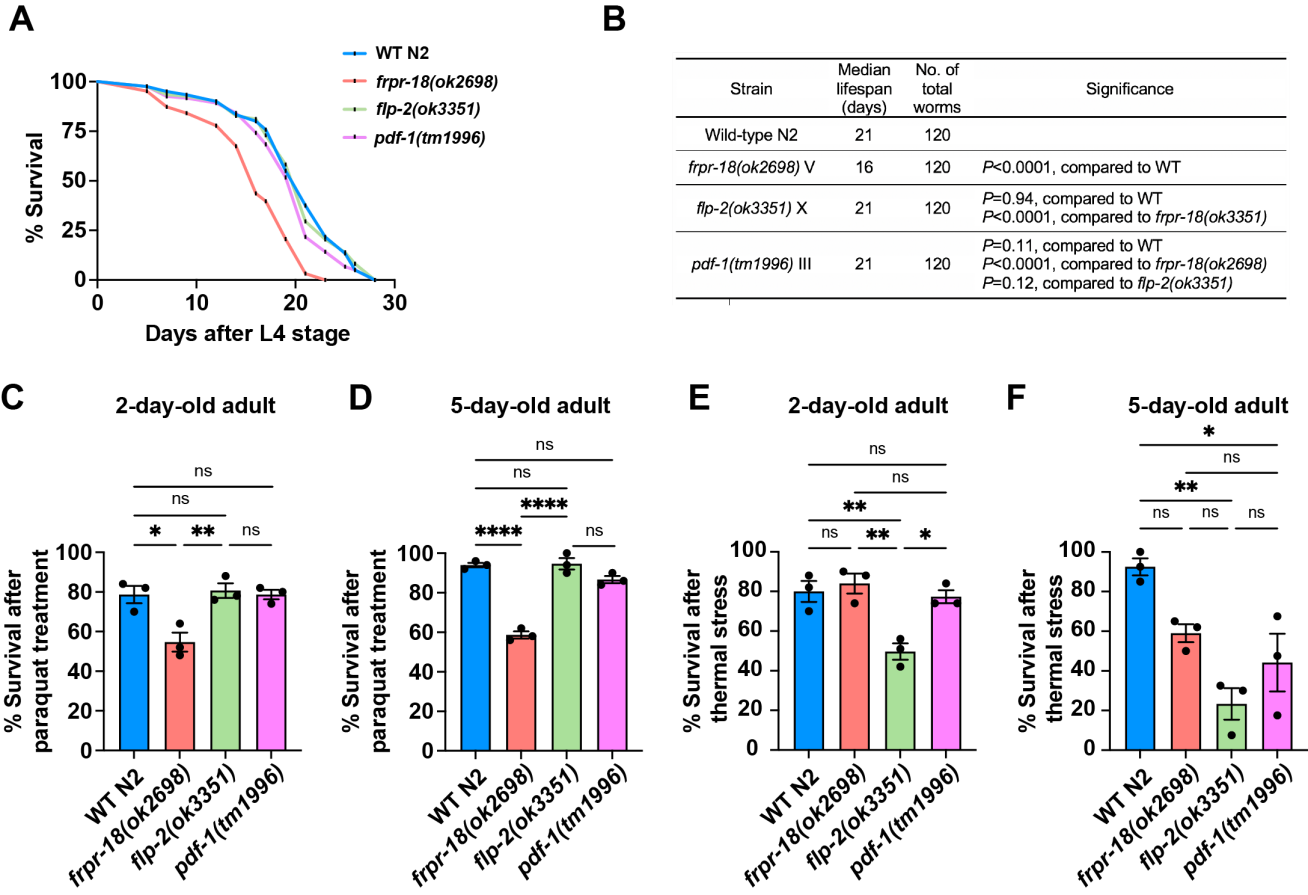
(A) Lifespan analysis of WT N2 , *frpr-18 ( ok2698 )* , *flp-2 ( ok3351 )* , and *pdf-1 ( tm1996 )* animals. (B) Summary of lifespan analysis. The asterisks in the "Strain" column indicate the genotype used for comparison. P values were analyzed by log-rank test. The results represent the total lifespan summary of triplicates. (C and D) Survival rate of animals cultured in 50 mM concentrations of paraquat for 48 hours at 2 and 5-day-old adult stages. (E and F) Survival rate after incubation at 35°C for 5 hours was measured after 24 hours in animals at 2 and 5-day-old adult stages. Each set of experiments included at least 50 animals. *P < 0.05, **P < 0.005, ***P < 0.005, ****P < 0.001; one-way ANOVA test with the Tukey multiple comparisons test. Data were presented as means ± SEM.

## Description

Neuropeptides are messengers synthesized and released by neurons (Nathoo et al., 2001) and well characterized by their critical roles in regulating post-synaptic target cells. Growing evidence suggests that neuropeptides are involved in the regulation of longevity in various organisms (Botelho and Cavadas, 2015; Chen et al., 2016b; Chiba et al., 2014; Miller et al., 2020; Savini et al., 2022; Shao et al., 2016; Zullo et al., 2019) . FRPR-18 is an orexin/hypocretin receptor ortholog and has been identified as an FLP-2 receptor to regulate arousal behavior (Chen et al., 2016a; Frooninckx et al., 2012; Mertens et al., 2005) . In mammals, orexin signaling decreases with age, and deficiency leads to reduced physical activity and obesity even if the calorie consumption is lower than its wild-type counterpart, suggesting its potential role in healthspan (Nixon et al., 2015). The FMRFamide-like peptide FLP-2 regulates the rhythmic pattern of locomotion quiescence and arousal, where its secretion is enhanced by PDF-1 neuropeptide signaling in reciprocal positive feedback (Chen et al., 2016a). FLP-2 neuropeptides also act as a signal expressed in a small group of sensory neurons and systematically coordinate the mitochondrial unfolded protein response (mitoUPR) in peripheral tissues in response to mitochondrial stress in neurons where it is expressed (Bar-Ziv et al., 2020; Shao et al., 2016) . Given that mitoUPR plays a key role in regulating the lifespan of animals with mitochondrial abnormalities, the role of FLP-2 signaling in the organismal lifespan has been intriguing (Durieux et al., 2011; Wu et al., 2018) . Shao et al. reported that FLP-2 overexpression in certain sensory neuron circuits is sufficient to enhance mitoUPR in peripheral tissues but does not alter the organismal lifespan (Shao et al., 2016) . In contrast, a recent study shows that FLP-2 overexpression extends the adult lifespan by regulating the secretion of one of the insulin-like peptides, INS-35 , in the intestine (Kageyama et al., 2022) . The lifespan of the loss-of-function condition for FLP-2 and its receptor FRPR-18 remains unclear. Thus, the role of FLP-2 / FRPR-18 signaling in regulating longevity and aging remains to be further clarified.

To investigate the role of FLP-2 and its receptor FRPR-18 in organismal aging, we conducted experiments to compare the lifespan and healthspan of *frpr-18 ( ok2698 )* null mutant strains with N2 wild-type control animals. Our findings indicate that the *frpr-18 ( ok2698 )* mutant had a shorter lifespan compared to the control. However *, flp-2 ( ok3351 )* null mutants exhibited similar longevity to N2 wild-type control animals ( Figure 1A and 1B). Next, we evaluated healthspan parameters, including stress tolerance against thermal and ROS, which could be uncoupled with longevity in some mutant conditions (Bansal et al., 2015) . We found that consistent with its effect on longevity, *frpr-18* mutants showed reduced tolerances against ROS induced by 50 mM paraquat treatment and thermal stress induced by culturing animals at 35 ˚C for 5 hours. Although *flp-2* null mutants showed a normal lifespan, they exhibited decreased survivability in the thermal assay, in both young adults and older adults (Day-5 adult stage). In contrast, *flp-2* mutants displayed normal tolerance to paraquat. We also tested the lifespan and stress tolerance of loss-of-function mutants of *pdf-1* , which has been shown to increase *flp-2* secretion in a reciprocal positive feedback manner. In agreement with the *flp-2* null mutant results, *pdf-1* null mutants also exhibited a normal lifespan ( Figure 1A and 1B), and stress tolerance against paraquat ( Figure 1C and 1D). However, it showed a decreased survivability against thermal stress at the 5-day-old adult stage ( Figure 1E and 1F).

Our results showed that *frpr-18 ( ok2698 )* mutants have alterations in lifespan and stress resistance. *flp-2 ( ok3351 )* mutants had a normal lifespan compared to WT control worms, suggesting that FRPR-18 could regulate lifespan independently of FLP-2 or through alternative compensatory ligands. Thus, it can be inferred that *frpr-18* regulates lifespan and stress tolerance in *C. elegans* through a complex and multifaceted mechanism, which involves numerous neuropeptide-signaling pathways. Previous studies reveal that FLP-2 loss is sufficient to result in a depletion of mitoUPR activation and abnormal arousal behavior (Shao et al., 2016) , suggesting that the depletion of FRPR-18 may also regulate health and longevity through a previously unknown biological function of FRPR-18 . However, we could not rule out the possibility that our results were affected by other background mutations as we used only one allele for each gene. Further experiments using another mutant allele, RNA interference, and rescue experiments are required.

## Methods

All *C. elegans* strains, including wild-type N2 , and mutant strains *flp-2 ( ok3351 )* X, *frpr-18 ( ok2698 )* V, and *pdf-1 ( tm1996 )* III, were maintained on Nematode Growth Medium (NGM) plates at 20°C, fed with OP50 Escherichia coli bacteria seeded onto each plate (Brenner, 1974; Stiernagle, 2006) . Synchronization of age was achieved by isolating eggs from adult worms and transferring L4 stage animals onto fresh 9-centimeter NGM plates two days before experimentation for Day-2-young adult experiments, while Day-2-worms were picked 3 days before Day 5 adult experiments. Floxuridine (FUDR) was added to the plates until the sixth day of the experiment to prevent reproduction and ensure no new nematodes were introduced into the populations (Gandhi et al., 1980) . Animals were transferred to unseeded NGM plates immediately before experimentation.

For the lifespan assay **,** the age-synchronized N2 worms for lifespan assay were done as described (Amrit et al., 2014; Rathor and Pandey, 2018) . Briefly, 35-40 L4 animals of each strain were transferred to three separate NGM agar plates spotted with E. coli OP50 . Additionally, 50 μM 5-Fluoro-2′deoxyuridine (FUdR) was added to the NGM plates to inhibit progeny growth, and the worms were transferred every 2-3 days to new OP50 seeded NGM plates. The lifespan of each strain was measured over the course of 28 days by counting and recording the number of worms that were alive and dead every few days while transferring the living worms into new plates. Thermal stress assays were done as described (Bar-Ziv et al., 2020b; Hyun et al., 2021) . The thermal stress treatments were performed in an incubator set to 35°C. Animals of each strain were developed and grown on NGM plates at 20°C until they reached the day-2 and day-5 adult stages. On day 2 and day 5, 50 animals from each strain were exposed to a 35°C incubator for 5 hours in three unseeded NGM plates. After 5 hours, fresh OP50 was added to the NGM plates after removal from the incubator to sustain the animals until all measurements were taken and kept at 20°C. The survival of animals was evaluated after 24 hours, and animals that failed to respond to a platinum wire touch were scored as dead. All experiments were performed in triplicate to ensure the reproducibility and reliability of the results. For the oxidative stress assays, paraquat was used to induce stress (Bar-Ziv et al., 2020b; Hyun et al., 2021) . Age-synchronized animals from each strain were raised from the L1 to the day 2-old adult stage on OP50 -seeded NGM plates. On day 2, 50 animals from each strain in triplicates were exposed to a 96-well plate containing a concentration of 50mM paraquat in a total volume of 300 microliters per well in M9 buffer. The worms were incubated at 20°C and viability was scored after 48 hours. All experiments were performed in triplicate to ensure the reproducibility and reliability of the results.
